# Mapping spoken language and cognitive deficits in post-stroke aphasia

**DOI:** 10.1016/j.nicl.2023.103452

**Published:** 2023-06-12

**Authors:** Haya Akkad, Thomas M.H. Hope, Charlotte Howland, Sasha Ondobaka, Katerina Pappa, Davide Nardo, John Duncan, Alexander P. Leff, Jenny Crinion

**Affiliations:** a Institute of Cognitive Neuroscience, University College London, UK; b Wellcome Centre for Human Neuroimaging, University College London, UK; c Institute of Neurology, University College London, UK; d MRC Cognition and Brain Sciences Unit, University of Cambridge, UK; e Department of Education, University of Roma Tre, Italy; f Department of Experimental Psychology, University of Oxford, UK; g Imaging Centre of Excellence, University of Glasgow, UK

**Keywords:** Stroke, Aphasia, Cognition, Language production, Lesion-symptom mapping

## Abstract

Aphasia is an acquired disorder caused by damage, most commonly due to stroke, to brain regions involved in speech and language. While language impairment is the defining symptom of aphasia, the co-occurrence of non-language cognitive deficits and their importance in predicting rehabilitation and recovery outcomes is well documented. However, people with aphasia (PWA) are rarely tested on higher-order cognitive functions, making it difficult for studies to associate these functions with a consistent lesion correlate. Broca’s area is a particular brain region of interest that has long been implicated in speech and language production. Contrary to classic models of speech and language, cumulative evidence shows that Broca’s area and surrounding regions in the left inferior frontal cortex (LIFC) are involved in, but not specific to, speech production. In this study we aimed to explore the brain-behaviour relationships between tests of cognitive skill and language abilities in thirty-six adults with long-term speech production deficits caused by post-stroke aphasia.

Our findings suggest that non-linguistic cognitive functions, namely executive functions and verbal working memory, explain more of the behavioural variance in PWA than classical language models imply. Additionally, lesions to the LIFC, including Broca’s area, were associated with non-linguistic executive (dys)function, suggesting that lesions to this area are associated with non-language-specific higher-order cognitive deficits in aphasia. Whether executive (dys)function – and its neural correlate in Broca’s area – contributes directly to PWA’s language production deficits or simply co-occurs with it, adding to communication difficulties, remains unclear. These findings support contemporary models of speech production that place language processing within the context of domain-general perception, action and conceptual knowledge. An understanding of the covariance between language and non-language deficits and their underlying neural correlates will inform better targeted aphasia treatment and outcomes.

## Highlights

• People with aphasia show impairment in non-linguistic cognitive functions.
• Damage to Broca’s area is associated with non-linguistic executive (dys)function.
• Broca’s area may not be a language-specific region as previously thought.

## 1 Introduction

While language impairment is the defining consequence of post-stroke aphasia, the presence of co-occurring impairments in other cognitive domains has been well documented (Fucetola et al., 2009, Helm-Estabrooks, 2002, Murray, 2012, El Hachioui et al., 2014, Marinelli et al., 2017, Ramsey et al., 2017, Schumacher et al., 2019). Despite this, People With Aphasia (PWA) rarely receive extensive cognitive assessment, meaning data on individual cognitive skills in this patient population is scarce. Evidence suggests that executive functions may be impaired in post-stroke aphasia, but the relationship between language and executive functions is difficult to tease apart (see Fedorenko, 2014) and studies have not been able to converge on the underlying lesions correlates of executive functions in PWA (Mirman and Thye, 2018).

Deficits in non-language specific cognitive domains have consistently been shown to be predictive of certain aspects of language function recovery in post-stroke aphasia. Marinelli and colleagues (Marinelli et al., 2017) examined language and cognitive function in 189 PWA and found more severe language deficits to be associated with more severe cognitive impairments. Other studies have investigated executive functions in PWA and consistently found impaired inhibition, working memory or cognitive flexibility (Murray, 2012, Frankel et al., 2007, Fridriksson et al., 2006, Jefferies et al., 2008, Lee and Pyun, 2014, Vallila-Rohter and Kiran, 2013). A better understanding of language processing in the context of other domain-general cognitive functions is important for clinical management and rehabilitation. In fact, a series of aphasia therapy studies emphasise that cognitive abilities, particularly executive functions and verbal short-term memory, play an important role in driving recovery outcomes (Fillingham et al., 2005, Fillingham et al., 2006, Fillingham et al., 2005, Conroy et al., 2009, Mirman et al., 2015, Lambon Ralph et al., 2010, Yeung and Law, 2010, Snell et al., 2010, Sage et al., 2011, Dignam et al., 2017, Lacey et al., 2017, Schumacher et al., 2020).

While studies have highlighted the impact of cognition on aphasia rehabilitation and recovery, few have explored the contribution of individual cognitive skills and the relationship to underlying lesion pattern. The neural basis of aphasia is commonly explored by linking behavioural assessment with brain lesion data. This has resulted in some distinct brain-behaviour relationships for various language domains, however studies have not been able to converge on a consistent lesion correlate of higher-level executive functions (Mirman and Thye, 2018), either because non-language assessments were not included (Mirman et al., 2015, Kümmerer et al., 2013) or were only included in a limited scope (Butler et al., 2014, Halai et al., 2017, Tochadse et al., 2018) though see Lacey et al. (2017). More recently, the neural correlates of non-language cognitive domains in aphasia have been explored by Schumacher et al., 2019, Schumacher et al., 2020, Alyahya et al., 2020, whose findings are discussed below.

Executive functions and language are closely linked in both brain and behaviour. Behaviourally, cognitive control and working memory have long been known to support language processing (Gordon et al., 2002, Novais-Santos et al., 2007, January et al., 2009, Fedorenko, 2014). Neurally, both executive functions and language robustly engage regions within the left frontal cortex (Kaan and Swaab, 2002, Novick et al., 2005). This makes it challenging to functionally dissociate anatomical correlates of the two domains. Of particular relevance is the function of Broca’s area and the left inferior frontal cortices (LIFC). Damage to Broca’s area, which encompasses cytoarchitecturally defined Brodmann’s area BA 44 and BA 45 of the left posterior inferior frontal gyrus (LpIFG) (Ardila et al., 2016, Papitto et al., 2020) commonly results in anomia, which has led people to believe that Broca’s area within the LpIFG play a causal role in language production. However, research in more recent years challenges this notion. The current view is that long-term speech production outcome following left inferior frontal damage is best explained by a combination of damage to Broca’s area and neighbouring regions including the underlying white matter (Gajardo-Vidal et al., 2021), which was also damaged in Paul Broca’s two historic cases (Dronkers et al., 2007), and that Broca’s area is not specialised for speech and language, but rather is part of a wider network of general cognitive processing that includes, but is not limited to language (Duncan, 2013, Duncan, 2010). Nevertheless, some argue that executive functions and language occupy nearby but distinct regions within the left frontal cortex (Fedorenko and Varley, 2016). To date, the brain areas required for speech production, and the type of aphasia that results from damage to the LIFC remains a topic of continued debate (SemMéd, 1906, Mohr et al., 1978, Alexander et al., 1990, Lorch, 2008, Fridriksson et al., 2015, Tremblay and Dick, 2016).

When assessing cognitive abilities, it is important to consider that cognition is a multidimensional construct broadly comprising five general domains, including language, attention, memory, executive functions and visuo-spatial skills (Helm-Estabrooks, 2002), with each domain containing distinct components. Using composite or general scores risks reducing the sensitivity of the cognitive measure. Schumacher and colleagues (Schumacher et al., 2019) recently demonstrated the importance of this by using a detailed non-verbal neuropsychological assessment to show that brain regions involved in particular components of the attention and executive functions domains contribute to the abilities of adults with a wide range of aphasia types. Lacey and colleagues (Lacey et al., 2017) showed that executive functioning explains considerable variance in language abilities of PWA. Schumacher et al. (Schumacher et al., 2020) recently showed that variance in functional communication abilities in PWA can be almost entirely explained by patients’ verbal short-term memory. Another study used extensive assessments of attention to show that different aspects of attention differentially predict language function in aphasia (Murray, 2012). Finally, studies that have explored the role of cognition in aphasia have typically involved a sample of diverse aphasia types and severity. While this is pertinent to capturing the incidence of cognitive impairment in the general aphasic population, the wide variability of aphasia subtypes can confound analyses of the links between domain-general cognitive impairment and any particular aphasic subtype or symptom.

Here, we investigated the behavioural and neural correlates of an extensive battery of language and non-language cognitive functions in a sample of 36 adults with post-stroke aphasia, who had long-term speech production deficits (anomia). Anomia is the most common symptom of post-stroke aphasia and manifests as difficulty in word retrieval when naming common objects (Laine and Martin, 2013). We collected a comprehensive behavioural battery containing language measures and an extensive assessment of individual cognitive domains. The participants in this study had relatively intact comprehension and no speech apraxia. The behavioural data were analysed using Principal Component Analysis (PCA) and their underlying lesion correlates were mapped using Voxel-Based Correlational Morphology (VBCM). PCA is a useful exploratory tool that can extract the underlying latent structure of a set of correlated variables – like scores in standardised assessments of post-stroke cognitive impairment. There has been increasing interest in interpreting these latent variables in terms of the potentially separable cognitive sub-systems underlying (often strongly correlated) task scores. This is typically done by correlating latent variables with the original scores: those scores that correlate more strongly with the latent variable are said to load on that latent variable. Here, following recent results, we employ varimax rotation to encourage greater sparsity, and thus interpretability, in those loadings (Butler et al., 2014, Halai et al., 2017, Tochadse et al., 2018).

In this study, we aimed to test whether speech production impairment in aphasia is associated with both language and non-language specific cognition and how this relates to correlates of brain damage. We investigated the underlying relationship between tests of individual cognitive skills, as well as language, in adults with chronic speech production deficits due to post stroke aphasia and identified the structural correlates of these cognitive and language features. We predicted that our analysis would reveal a latent, non-language specific cognitive component to speech production difficulties, which would be driven by damage to Broca’s area and surrounding regions in the left frontal cortices.

## 2 Material and methods

### 2.1 Participants

**Fig. 1:**
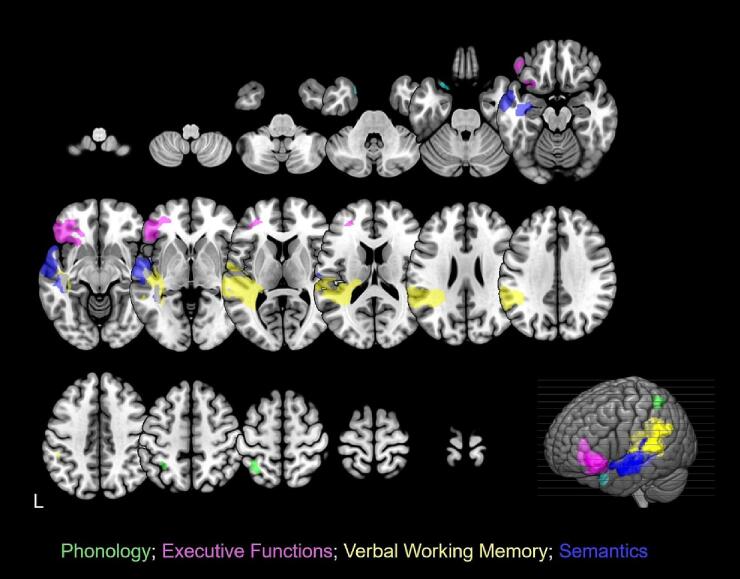
Structural correlates associated with each component from the combined PCA. Phonology: green; Executive Functions: magenta; Verbal Working Memory: yellow; Semantics: two distinct clusters in cyan and indigo. Clusters were obtained by applying a voxel-level threshold at p ≤ 0.001 and a family-wise error correction of p < 0.05 at cluster level. The lower right corner displays a rendered template brain (created in MRIcro-GL) showing the significant clusters projected to the left brain surface. (For interpretation of the references to colour in this figure legend, the reader is referred to the web version of this article.)

Thirty-six English speakers with chronic aphasia following a single left-hemisphere stroke participated in the study (see Fig. 1 for a lesion overlap map, Table 1 for demographic and clinical data). All were at least 12 months post-stroke and at the time of scanning and assessment, had normal hearing, normal or corrected-to-normal visual acuity and no previous history of significant neurological or psychiatric disease. Inclusion criteria were: (i) anomia as determined by the naming subtest of the Comprehensive Aphasia Test (Swinburn et al., 2004); (ii) good single word comprehension as indexed by an accuracy score>70% on the spoken word comprehension subtest of the Comprehensive Aphasia Test (Swinburn et al., 2004); (iii) relatively spared ability to repeat single monosyllabic words from the Psycholinguistic Assessments of Language Processing in Aphasia (Kay, 1992); (iv) absence of speech apraxia as determined by the Apraxia Battery for Adults (Dabul, 2000). Participants were excluded if they had any contraindications for scanning or any other significant neurological or psychiatric conditions. Informed consent was obtained from all participants in accordance with the Declaration of Helsinki and the study was approved by the Central London Research Ethics Committee, UK.

**Table 1:** Participant demographic and clinical data.

**ID**	**Gender**	**Age (years)**	**Education (years)**	**Time post-stroke (years)**	**Total lesion volume (cm^3^)**
01	M	55	16	11	61.53
02	M	56	11	7	160.80
03	M	71	13	2	42.76
04	M	55	11	9	57.24
05	M	71	16	2	78.36
06	F	51	13	13	43.42
07	M	47	13	12	161.81
08	F	66	16	18	83.18
09	M	61	16	4	63.56
10	M	44	16	3	38.14
11	F	44	17	1	29.49
12	F	70	11	12	8.93
13	M	70	11	29	117.55
14	M	69	16	7	171.71
15	M	73	11	11	71.31
16	F	45	11	3	63.53
17	F	53	11	5	22.25
18	F	55	13	5	1.51
19	M	40	17	8	163.75
20	M	64	13	24	308.18
21	M	42	17	1	65.80
22	M	74	16	11	164.53
23	M	63	16	25	156.91
24	M	64	16	10	348.23
25	M	60	16	11	94.32
26	F	60	11	6	223.29
27	M	75	11	12	112.55
28	F	50	16	3	130.60
29	M	64	11	8	240.39
30	M	29	13	6	78.61
31	F	81	10	16	99.38
32	M	60	13	20	403.11
33	M	65	13	14	387.17
34	M	82	13	34	152.61
35	M	58	16	8	239.29
36	M	39	17	2	95.70

Thirty-six participants. 10 Female, age range 29–82 years (mean: 59, SD: 12.5). Average time post-stroke was 10 years and average lesion volume was 131.7 cm^3^.

### 2.2 Neuropsychology

#### 2.2.1 Behavioural assessment

A comprehensive battery of language and non-language tests was administered to assess participants’ language and cognitive abilities (see supplementary material for all administered behavioural tests [Supplementary Table 1] and percentage of participants with impaired performance scores [supplementary Fig. 2]).

The language tests administered to assess speech production included the naming and repetition subtests of the CAT, the word/non-word repetition subtests from the Psycholinguistic Assessments of Language Processing in Aphasia subtests 8 and 9 (PALPA (Kay, 1992)), the Boston Naming Test (BNT (Kaplan, 1983)). The language assessments that captured other language functions included Pyramids and Palm Trees (PPT) (Howard and Patterson, 1992), other subtests from the CAT, and the reading tasks from the PALPA8.

The non-language cognitive assessments included the Cattel Culture Fair IQ Test (Cattell, 1960) (Scale 2, Form A), Rey-Osterrieth Complex Figure Test (Osterrieth, 1944), Digit Span tasks from the Wechsler Adult Intelligence Scale – Fourth Edition (WAIS-IV) (Wechsler, 1955), the trail making and card sorting subtests from the Delis-Kaplan Executive Functions System test (D-KEFS) (Delis, 2001), the Hopkins Verbal Learning Test (HVLT) (Brandt and Benedict, 2001) and the Children’s Sustained Attention to Response Task (cSART) (Robertson et al., 1997).

#### 2.2.2 Principal component analysis (PCA)

PCA is a useful exploratory tool that can extract the underlying latent structure of a set of correlated variables – like scores in standardised assessments of post-stroke cognitive impairment. There has been increasing interest in interpreting these latent variables in terms of the potentially separable cognitive sub-systems underlying (often strongly correlated) task scores. This is typically done by correlating latent variables with the original scores: those scores that correlate more strongly with the latent variable are said to load on that latent variable. Here, following recent results, we employ varimax rotation to encourage greater sparsity, and thus interpretability, in those loadings (Butler et al., 2014, Halai et al., 2017, Tochadse et al., 2018).

Participants’ scores on all assessments were entered into a PCA with varimax rotation (conducted with SPSS 26.0). Missing data was replaced by the mean. However, incidence of missing data was low, limited to two data points across the dataset (Supplementary Table 1). We had 55 variables and 36 cases. Factors with an eigenvalue ≥ 1.0 were extracted then rotated. After orthogonal rotation, the factor loadings of each test allowed interpretation of what cognitive-language primary process was represented by that factor (Table 2). Individual participants’ scores on each extracted factor were then used as behavioural covariates in the neuroimaging analysis.

**Table 2:** Loadings of behavioural assessments on rotated PCA factors.

	Factor 1 Phonology	Factor 2 Executive Functions	Factor 3 Verbal Working Memory	Factor 4 Semantics
PALPA9 Repetition - Words (LILF)	**0.898**	0.089	0.064	0.190
PALPA9 Repetition - Words (LIHF)	**0.859**	−0.125	0.196	0.204
PALPA9 Repetition - Words (HILF)	**0.829**	0.189	0.054	0.055
PALPA9 Repetition Non-Words	**0.826**	0.045	0.299	0.135
PALPA8 Repetition Non-Words	**0.792**	−0.024	0.308	0.106
PALPA9 Repetition - Words (HIHF)	**0.778**	0.108	0.020	0.099
CAT Repetition - Words	**0.696**	0.075	0.003	0.404
CAT Repetition - Non-Words	**0.620**	0.227	0.180	0.019
CAT Comprehension - Spoken Words	**0.606**	0.269	−0.096	−0.099
CAT Repetition - Complex Words	**0.580**	0.042	0.252	0.265
DKEFS Card Sorting: Free Description	0.036	**0.892**	0.073	0.212
DKEFS Card Sorting: Correct Sorts	0.119	**0.888**	0.060	0.179
DKEFS Card Sorting: Recognition	0.089	**0.841**	0.207	0.219
DKEFS Card Sorting: Perceptual Sorts	0.073	**0.836**	0.178	0.225
DKEFS Card Sorting: Verbal Sorts	0.177	**0.599**	−0.052	0.141
WAIS Forward Digit Span	0.286	0.014	**0.870**	0.098
WAIS Backward Digit Span	0.013	0.014	**0.791**	0.000
CAT Repetition - Digit String	0.121	0.229	**0.776**	0.173
CAT Repetition - Sentences	0.233	−0.004	**0.650**	**0.526**
PALPA8 Reading - Non-Words	0.195	0.228	**0.628**	0.382
CAT Spoken Picture Description	0.276	0.241	**0.537**	0.330
CAT Reading - Non-Words	0.310	0.378	**0.506**	0.324
Boston Naming Test	0.123	0.440	−0.018	**0.771**
CAT Reading - Words	0.298	0.243	0.314	**0.748**
CAT Naming - Objects	0.400	0.055	0.099	**0.713**
CAT Naming - Actions	0.136	0.281	0.170	**0.646**
CAT Reading - Complex Words	0.273	0.049	**0.568**	**0.640**
CAT Reading - Function Words	0.088	0.265	0.268	**0.544**
CAT Writing to Dictation	0.161	0.290	0.399	**0.529**
CAT Comprehension -Written Sentences	0.138	0.139	0.372	**0.510**

Factor loadings > 0.5 are given in bold. PALPA = Psycholinguistic Assessments of Language Processing in Aphasia; LILF = Low Intelligibility Low Frequency, LIHF = Low Intelligibility High Frequency, HIHF = High Intelligibility High Frequency, HILF = High Intelligibility Low Frequency. CAT = Comprehensive Aphasia Test. DKEFS = Delis-Kaplan Executive Functions System. WAIS = Wechsler Adult Intelligence Scale. Tests with very low loadings (<0.001) do not appear in this table, however, all tests were included in the analysis.

### 2.3 Neuroimaging

#### 2.3.1 MR imaging acquisition and analysis

Whole-brain imaging was performed on a 3 T Siemens TIM-Trio system (Siemens, Erlangen, Germany) at the Wellcome Centre for Human Neuroimaging. Structural (T1-weighted) MRI images were normalised using Statistical Parametric Mapping software (SPM12) running under Matlab 2015a (MathWorks, Natick, MA). Lesion images were defined by the Automatic Lesion Identification toolbox (ALI) (Seghier et al., 2008), employing a variant of the unified segmentation algorithm (Ashburner and Friston, 2005), optimised for use in the focally damaged brain.

Structural MRI scans were pre-processed with Statistical Parametric Mapping software (SPM12: Wellcome Trust Centre for Neuroimaging, https://www.fil.ion.ucl.ac.uk/spm/). The images were normalised into standard Montreal Neurological Institute (MNI) space using a modified unified segmentation–normalisation procedure optimised for focal lesioned brains (Seghier et al., 2008). Data from all participants were entered into the segmentation–normalisation. This procedure combines segmentation, bias correction and spatial normalisation through the inversion of a single unified model (see  (Ashburner and Friston, 2005) for more details) (Ashburner and Friston, 2005). In brief, the unified model combines tissue class (with an additional tissue class for abnormal voxels), intensity bias and non-linear warping into the same probabilistic models that are assumed to generate subject-specific images. Images were then smoothed with an 8 mm full-width-half-maximum (FWHM) Gaussian kernel and used in the lesion analyses described below. The lesion of each participant was automatically identified using an outlier detection algorithm, compared to healthy controls, based on fuzzy clustering. Voxel values in these regions range from 0 to 1, with higher values indicating greater evidence that the voxel is damaged, and evidence is derived by comparing tissue intensity in each voxel to intensities from a population of neurologically normal controls. The default parameters were used. The images generated for each participant were individually checked and visually inspected with respect to the original scan and were used to create the lesion overlap map in Fig. 1. We selected the Seghier et al. (2008) method as it is objective and efficient for a large sample of lesions (Wilke et al., 2011).

#### 2.3.2 Lesion-Symptom mapping

For lesion-symptom mapping, we used the fuzzy lesion images as described above and correlated these with PCA factor scores using a voxel-based correlational methodology (VBCM) (Tyler et al., 2005), a variant of voxel-lesion symptom mapping (VLSM) (Bates et al., 2003). We used VBCM because this approach i) has the virtue of preserving the continuous nature of both behavioural and neural indices i.e., does not require a binary classification of the intact/lesioned brain to be marked, as in the case of VLSM, and ii) replicates previous methodology using varimax-rotated PCA in aphasia (Schumacher et al., 2019, Butler et al., 2014, Halai et al., 2017, Tochadse et al., 2018, Alyahya et al., 2020), aiding data comparisons within the field.

The VBCM analysis of PCA factors was conducted in SPM12 running on Matlab 2019b. The analysis used the four continuous multidimensional predictors of the PCA factor scores, which are necessarily uncorrelated (orthogonal) with one another; these were entered simultaneously as continuous behavioural covariates. The outcome of the analysis therefore denotes which voxels’ variation in tissue concentration corresponds to the unique variance in a given principal component, while controlling for variation in the other components in the analysis. In order to ensure that the results were not merely attributable to lesion size, each participants' lesion volume was calculated from the lesion identified by the automated lesion identification method (Seghier et al., 2008) and this was entered as a covariate in the VBCM. All analyses were performed with and without a correction for lesion volume. All anatomical labels were based on the Harvard–Oxford atlas in MNI space.

## 3 Results

### 3.1 Neuropsychological profiles and principal language-cognitive factors

The rotated PCA produced a four-factor solution which accounted for 55% of variance in participants’ performance (F1 = 28.6%; F2 = 10.6%; F3 = 8.3%; F4 = 7.1%). The loadings of each of the different behavioural assessments on each of the factors are given in Table 2 (for individual participants’ scores on each factor and percentage of participants with impaired language and non-language scores, see Supplementary Table 1 and supplementary Fig. 2 respectively). Tasks that tapped into input and output phonology (e.g. word and non-word repetition) loaded heavily on Factor 1, as such we refer to this factor as ‘Phonology’. Factor 2 was interpreted as ‘Executive Functions’, as assessments that loaded most heavily on it tapped into non-verbal cognitive processes (e.g. problem solving and concept formation). Assessments that loaded on Factor 3 were those requiring speech output (e.g. composite picture description) and online maintenance and use of auditory inputs (e.g. digit span, sentence repetition) along with phonological skills (e.g. reading aloud non-words), hence we refer to this factor as ‘verbal working memory’. Finally, Factor 4 was interpreted as ‘Semantics’, the assessments that loaded on this factor were more diverse but primarily required processing of meaning (e.g. picture naming and comprehension of written sentences).

### 3.2 The neural basis of performance in chronic stroke aphasia

#### 3.2.1 Voxel-based correlational morphology of principal component analysis factors

**Fig. 2:**
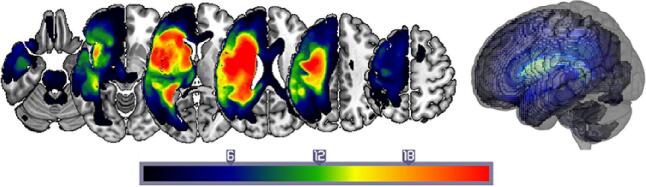
Lesion overlap map. A lesion overlap map for the 36 S anomic participants. Colour scale represents frequency of regional brain damage (hot-body scale with red indicating most frequently damaged brain regions i.e., >18 patients, while dark blue < 6 patients with damage to these regions). Results are shown overlaid on the MNI template brain, created in MRIcro-GL. (For interpretation of the references to colour in this figure legend, the reader is referred to the web version of this article.)

The VBCM results are shown in Fig. 2 and Table 3. Each map displays where tissue damage covaries uniquely with a given factor score, where the factors are necessarily uncorrelated with one another. Results are thresholded at p ≤ 0.001 voxel-level and p < 0.05 FWE corrected at cluster-level.

**Table 3:** Neural correlates for omnibus PCA factors.

**Principal Component**	**Location**	**Extent (voxels)**	**Z**	**MNI co-ordinates**		
				**x**	**y**	**z**
**F1 (Phonology)**	Left Superior Parietal Lobe	175	4.16	−34	−54	58
**F2 (Executive Functions)**		1563				
	Left Inferior Frontal Gyrus (Pars Triangularis)		3.93	−44	36	2
	Left Inferior Frontal Gyrus (Pars Orbitalis)		3.59	−40	42	−8
	Left Middle Frontal Gyrus (Dorsolateral Prefrontal Cortex)		3.56	−30	44	4
**F3 (Verbal Working Memory)**		5262				
	Left Posterior Superior Temporal Gyrus		4.49	−58	−28	6
	Left Superior Longitudinal Fasciculus		4.94	−38	−44	18
	Left Posterior Thalamic Radiation		5.35	−36	−46	2
**F4 (Semantics)**		209				
	Left Superior Temporal Pole		4.82	–22	10	−24
	Left Middle Temporal Pole		4.47	−20	12	−34
		2343				
	Left Superior Temporal Gyrus		4.14	−64	−10	−4
	Left Middle Temporal Gyrus		4.14	−62	−14	−12

Only clusters with cluster-level FWEc p < 0.05 are shown in the table.

Performance on the phonological factor was uniquely correlated with a cluster of voxels in the left parietal lobe, with peak voxels in the left superior parietal lobe and intraparietal sulcus. The cluster also included voxels in the left inferior parietal lobule.

Performance on the semantic factor was uniquely related to two clusters in the left hemisphere with peak voxels in the superior/ middle temporal pole and superior/middle temporal gyrus. The clusters also included voxels across the left insula.

Performance on the verbal working memory factor was uniquely related to a large cluster of voxels in the left hemisphere, with peak voxels in the posterior superior temporal gyrus, the superior longitudinal fasciculus and posterior thalamic radiation. Note that VBM and structural MRI scans are not optimised for the detection of white matter fibre bundles. As such, any interpretation of white matter tracts should be made with caution. The cluster also included voxels within left Heschl’s gyrus and the hippocampus.

Performance on the executive functions factor was uniquely related to a cluster of voxels in the left frontal lobe, with peak voxels in the left dorsolateral prefrontal cortex and the left inferior frontal gyrus (LIFG, pars orbitalis and pars triangularis). To further probe the relationship between damage to Broca’s area within the LIFG and linguistic and non-linguistic executive functions in our cohort of PWA, we ran a post-hoc ROI analysis specifically testing whether lesions to Broca’s area were driving impairment in verbal or non-verbal executive functions. To do this, we calculated lesion load in BA44 and BA45 (Broca’s area) for each participant and ran a Pearson’s correlation against the verbal (linguistic) and perceptual (non-linguistic) scores that loaded on this executive functions PCA factor (see Table 2 for a breakdown on the individual executive function scores that loaded on this PCA factor). Lesion load was calculated as the sum of lesioned voxels that fell within BA44/45 (see section 2.3.1 for how lesioned voxels were estimated). Lesion load in Broca’s area correlated negatively with both linguistic (r = -0.32, p = 0.056) and non-linguistic scores (r = -0.37, p = 0.025), but this relationship was only significant for the non-linguistic score.

#### 3.2.2 Controlling for lesion size, age and time post-stroke

Given that 1) larger lesions will encompass more brain regions, 2) some regions are more susceptible to age-related atrophy and 3) time post-stroke can influence brain plasticity and recovery, we controlled for lesion volume, age and time post-stroke in subsequent lesion-symptom analyses.

Each participant’s lesion volume was calculated from the lesion identified by the modified segmentation-normalization procedure (see ‘Materials and methods’ section). For the PCA factors, lesion volume correlated relatively weakly with the phonology factor (r = 0.137, p = 0.426), the auditory working memory factor (r = -0.318, p = 0.059) and semantic factor (r = -0.313, p = 0.063), and slightly more strongly with the executive-functions factor (r = -0.426, p = 0.10).

Including age and time post-stroke in the VBCM model with the PCA factor scores did not alter the pattern of results obtained. All participants were in the chronic stage, which might be why time post-stroke, an often important covariate, had no substantive effect on the results. However, including lesion volume in the model reduced the significance of the executive functions measure, which only reached suprathreshold at voxel-level p < 0.05 and FWEc cluster-level p < 0.05, but did not alter the pattern of results in the remaining 3 PCA factors. As previously mentioned, the executive functions component correlated with tissue damage in the left inferior frontal cortex (LIFC); as a common region of damage following left MCA stroke (Phan et al., 2005), high covariance between LIFC tissue integrity and total lesion volume is expected.

## 4 Discussion

The aim of the current study was to investigate the presence of latent cognitive factors that might explain the variance in aphasic language production abilities and how this relates to underlying lesion patterns. We conducted an extensive language and non-language neuropsychological assessment in a sample of thirty-six PWA with long-term language production deficits. Our results replicate and extend work on the neural correlates of higher-level cognitive functions in PWA and their role in language production. We show that (i) the variance underlying language and non-language test performance was best captured by four orthogonal components, two non-linguistic cognitive components (executive functions and verbal working memory) and two linguistic processing components (phonology and semantics) (Table 2); (ii) brain-behaviour relationships revealed separable neural correlates for each component in line with previous studies and showed that lesions to the left inferior frontal cortex (LIFC) are associated with non-linguistic executive dysfunction (Fig. 2, Table 3), suggesting that these regions may be involved in, but are not specific to, language production.

The neural correlates associated with the two language components were supported by previous literature. The phonological component explained the largest proportion of behavioural variance in our group of anomic adults. Scores on this component, which in our study loaded principally on tests of single word and non-word repetition, uniquely correlated with tissue damage in the left superior parietal lobe and intraparietal sulcus (Fig. 2). Previous work shows that impaired speech repetition following left hemisphere stroke is associated with left parietal lobe damage (Fridriksson et al., 2010). However, unlike our results, the reported regions were in the inferior parietal lobe. More recent studies that have used a similar approach to ours, with a combined rotated PCA and VBCM in people with aphasia reported a phonology component uniquely related to left temporo-parietal regions (Schumacher et al., 2019, Butler et al., 2014, Halai et al., 2017, Alyahya et al., 2020). It is important to note that the phonology component in those studies also loaded on tests of naming and verbal working memory, as well as repetition, whereas our phonology component was specific to input/output phonology and loaded heavily on tests of single word and non-word repetition. The superior parietal lobe and intraparietal sulcus have been implicated in verbal short-term maintenance during language repetition (see Majerus, 2013 for a review (Majerus, 2013)). In healthy individuals, these regions are engaged with increased load (e.g. novel or unfamiliar phoneme sequences, multiple verbal stimuli) during language repetition. It is possible that in our cohort of PWA, who have long-term speech production deficits (anomia), errors in word/ non-word repetition may be due to difficulty with verbal short-term maintenance of phonological input. The semantic component explained the least amount of behavioural variance in our sample. Scores on this factor loaded on tests of naming, reading and written comprehension and uniquely correlated with regions in the left superior/ medial temporal pole and the left superior/ medial temporal gyrus (Fig. 2). This supports recent findings that extend the temporal regions implicated in semantic processing (Jackson, 2021).

Higher cognitive functions, namely executive functions and verbal working memory, independently explained a significant amount of behavioural variance in PWA with chronic language production deficits. Both have also been shown to be robust behavioural predictors of aphasia recovery outcomes (Fillingham et al., 2005, Fillingham et al., 2006, Fillingham et al., 2005, Conroy et al., 2009, Lambon Ralph et al., 2010, Yeung and Law, 2010, Snell et al., 2010, Sage et al., 2011, Dignam et al., 2017). During aphasia recovery, executive functions are argued to be important for the generation of semantic and phonological concepts to aid with word retrieval (Dignam et al., 2017) and to navigate other complex dynamics of human communication, while the integrity of general memory processes enables (re)learning and retention of linguistic knowledge during rehabilitation. Schumacher and colleagues (Schumacher et al., 2020) show that variance in functional communication abilities in PWA, as measured by the Amsterdam Nijmegen Everyday Language Test, can be almost entirely accounted for by patients’ verbal short-term memory. In our study, the verbal working memory component uniquely correlated with regions of tissue damage in the left posterior superior temporal gyrus, left superior longitudinal fasciculus, as well as Heschl’s gyrus and the hippocampus (Fig. 2). We note that VBM and structural MRI scans are not optimised for the detection of white matter fibre bundles and therefore, any interpretation of white matter tracts in this study are limited. This component captured abilities both in continuous (narrative) speech production (e.g, spoken picture description) and online maintenance of increasing auditory information (e.g. digit-span, sentence repetition). This replicates findings from Tochadse et al. (Tochadse et al., 2018) who report a similar neural correlate associated with auditory working memory in PWA.

Scores on the executive functions factor uniquely correlated with tissue damage in the left inferior frontal cortex (LIFC), including pars orbitalis and pars triangularis, and middle frontal gyrus (DLPFC) (see Fig. 2 for structural correlates and Table 3 for MNI co-ordinates). The LIFC results support and extends recent findings from ECoG and fMRI. Conner et al. (2019) used intracranial recordings to show that activity in pars triangularis and pars orbitalis is specifically engaged in object naming, compared to scrambled images, and shows stronger activity for words with high selectivity (number of possible correct responses). Ekert et al. (2021) used fMRI to show that pars orbitalis was most activated during object naming, compared to repetition of words and pseudowords. Our participants all had anomia, and by definition significant object naming deficits, however, our results show that lesions to Broca’s area (pars triangularis and pars opercularis) and pars orbitalis are associated with non-linguistic executive functions, namely concept formation and problem solving. This suggests that these regions within the LIFC support high-level planning and execution that is important for object naming, but not specific to language processing. These findings support the role of Broca’s area, and adjacent pars orbitalis in domain-general cognition and extend our understanding of the neural correlates of spoken language impairment in aphasia. This supports contemporary models of speech and language that suggest that language production may rely on the same process and neural systems that support other high-level action planning and execution (Botvinick, 2008, Hickok, 2012, Weiss et al., 2016). We show that in a group of PWA with chronic speech production deficits, lesions to Broca’s area and adjacent pars orbitalis within the LIFC are associated with non-linguistic executive (dys)function. This suggests that, while damage to Broca’s area and the LIFC commonly coincides with language impairment after stroke, lesions to this area appear to be driving non-language specific executive (dys)function. It remains unclear whether executive (dys)function – and its neural correlates in Broca’s area and surrounding LIFC – contributes directly to language production deficits in PWA or co-occurs with it, adding to their communication difficulties. Lesions to Broca’s area may lead to deficits in high-level executive functioning that either occurs independently of language function per se, or contributes directly to varying levels of language production impairment. This will likely depend on the pattern of damage to neighbouring regions of grey and white matter (Gajardo-Vidal et al., 20212021, Kimberg et al., 2007, Richardson et al., 2012, Inoue et al., 2014, Mah et al., 2014, Sperber and Karnath, 2017). Further work is necessary to improve our understanding of how the interaction between structural stroke anatomy and known functional networks affects the pattern of behavioural performance in PWA and the resulting communication challenges they face.

Behaviourally, the executive functions component loaded on tests of problem solving and concept formation as measured by the D-KEFS Card Sorting assessment. Card sorting assessments, including the D-KEFS and Wisconsin (Berg, 1948) tasks, appear to reliably engage executive functions and relate to damage in the left inferior frontal cortices (LIFC) and DLPFC in our group of aphasic adults. The neural correlates associated with our executive functions component show some overlap, namely pars triangularis and DLPFC, with a PCA component identified by Schumacher and colleagues (Schumacher et al., 2019), which the authors refer to as ‘inhibit-generate’. Their ‘inhibit-generate’ component captured abilities of idea generation, reasoning, problem solving and response inhibition in PWA and loaded on, amongst others, the D-KEFS card sorting test, as we used here. DLPFC is also reported by Lacey et al. (2017) as a neural correlate of their executive functions component which loaded on, amongst others, tests of planning, rule following and cognitive flexibility in PWA. Alyahya et al. (2020) also identify the middle frontal gyrus as a structural correlate of executive functions, specifically tests of abstract reasoning and rule following, in aphasic adults. Baldo et al. (2005) reported impairment on the Wisconsin Card Sorting Task in aphasic individuals, but not in adults with left-hemisphere brain damage without aphasia, suggesting that the card sorting task taps into executive functions that are necessary for effective language function. Consistent with this, Dignam et al. (2017) show that the D-KEFS Card Sorting assessment is predictive of successful anomia therapy outcomes. Collectively, these findings suggest that in PWA, card sorting tasks such as the D-KEFS, that we used here, are a sensitive measure of executive functions supporting language functioning. Not including these assessments of concept formation and problem-solving may be one of the reasons previous aphasia studies have struggled to find consistent associations between tests of executive functions and brain damage (Mirman et al., 2015, Kümmerer et al., 2013, Butler et al., 2014, Halai et al., 2017, Tochadse et al., 2018).

## 5 Conclusions

Our findings suggest that in people with chronic speech production deficits post-stroke, non-linguistic cognitive functions, namely executive functions and verbal working memory, explain more of the variance in long-term speech production deficits than classical models imply (Tremblay and Dick, 2016). Moreover, the LIFC, in particular Broca’s area and adjacent pars orbitalis, appear to be associated with non-linguistic executive functions, namely concept formation and non-verbal problem solving, suggesting that these regions may contribute to language production deficits in aphasia, but may not be language-specific regions as previously thought. It is important to note that our findings are limited by a small sample size and should be interpreted with caution. Nonetheless, they are supported by a recent body of work that 1) brings into question the modular, “language centric” perspective of the Classic “Wernicke-Lichtheim-Geschwind” model (Tremblay and Dick, 2016), 2) highlights the importance of non-linguistic cognitive domains and their neural correlates in aphasia; specifically, executive function, verbal working memory and attention (Murray, 2012, Schumacher et al., 2019, Lacey et al., 2017, Schumacher et al., 2020, Alyahya et al., 2020), and 3) shows that isolated damage to Broca’s area does not appear to contribute to long-term speech production outcome (Gajardo-Vidal et al., 2021). This does not necessarily imply that all PWA will have additional cognitive impairments, but that in those who do, higher-level executive functions may explain more of the behavioural variance in PWA than previously thought. A better understanding of the covariance between language and non-language deficits and their underlying neural correlates will inform more targeted aphasia treatment, tailored to an individual’s pattern of impairments. This may be in the form of neurostimulation targeting regions of domain-general cognition or by incorporating measures of non-linguistic cognitive function, such as concept formation and verbal working memory, to improve the accuracy of aphasia prediction models (Price et al., 2010, Hope et al., 2013, Hope et al., 2018, Yourganov et al., 2015).

## Data availability

The data described in this study is available to accredited researchers from J.C., on request.

## Funding

HA holds a doctoral fellowship funded by Brain Research UK (552175). This research was funded in part, by the Wellcome Trust [203147/Z/16/Z and106161/Z/14/Z J.C]. For the purpose of open access, the author has applied a CC BY public copyright licence to any Author Accepted Manuscript version arising from this submission. The funders had no participation in the design and results of this study.

## Declaration of Competing Interest

The authors declare that they have no known competing financial interests or personal relationships that could have appeared to influence the work reported in this paper.

## Data Availability

Data will be made available on request.
