# Supplementary material for: Mapping spoken language and cognitive deficits in post-stroke aphasia
Source: Neuroimage Clin. 2023 Jun 12;39:103452. doi: 10.1016/j.nicl.2023.103452 (PMC10275719; doi:10.1016/j.nicl.2023.103452)
Supplement: Supplementary data 1 [file mmc1.docx]

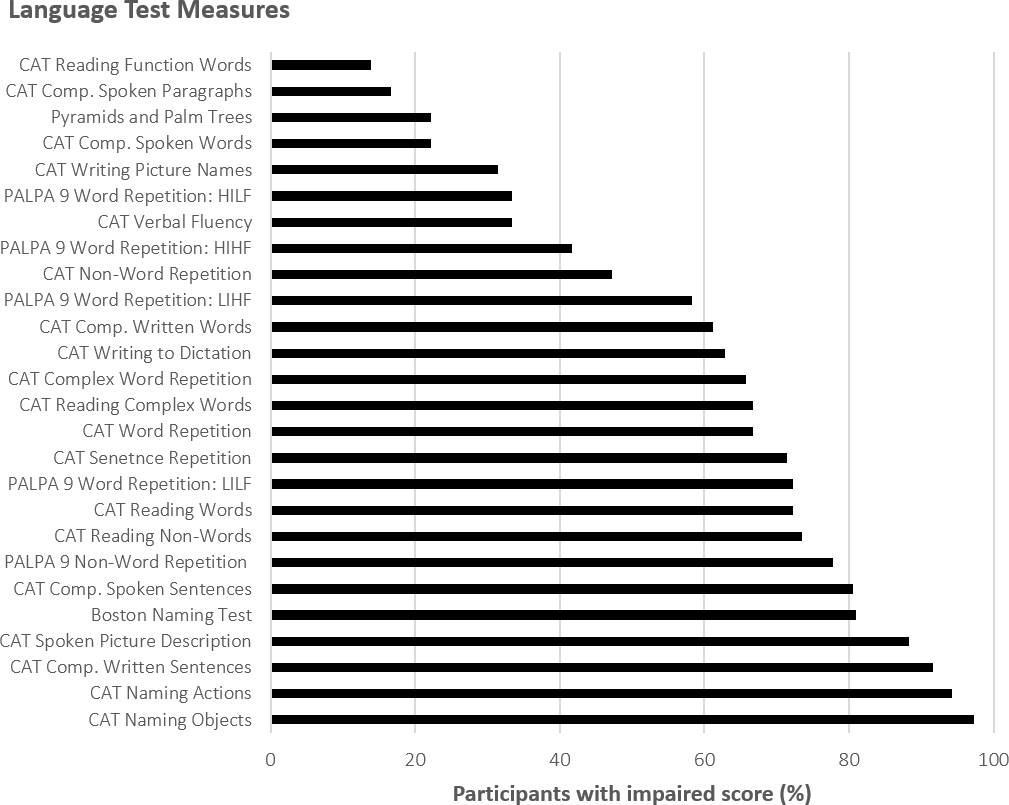


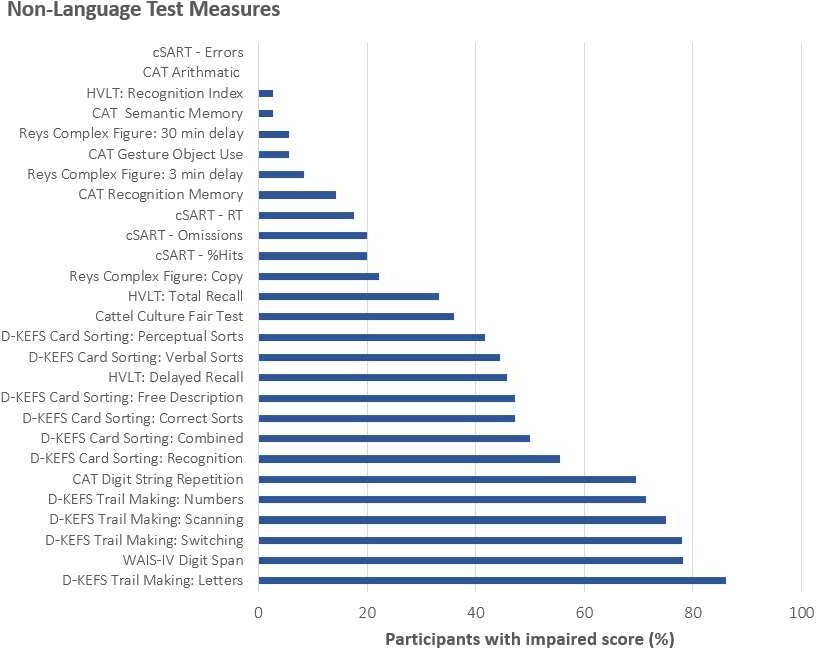


Supplementary Figure 1. Percentage of anomic participants with impaired performance on language and non-language tests. Impairment was determined based on a cut-off score defined by the test manual. Where this was not available, a cut-off was based on a score two standard deviations below the mean of a normative sample. Normative data for the Boston Naming test retrieved from Nicholas et al., 1989; Cattell's culture fare test (Tranter and Koutsaal, 2008). No cut-offs available for Raven's coloured progressive matrices and PALPA 8. See supplementary table 1 for individual participants' scores on behavioural assessments.

**Supplementary Table 1. Participants’ scores on the behavioural assessments**


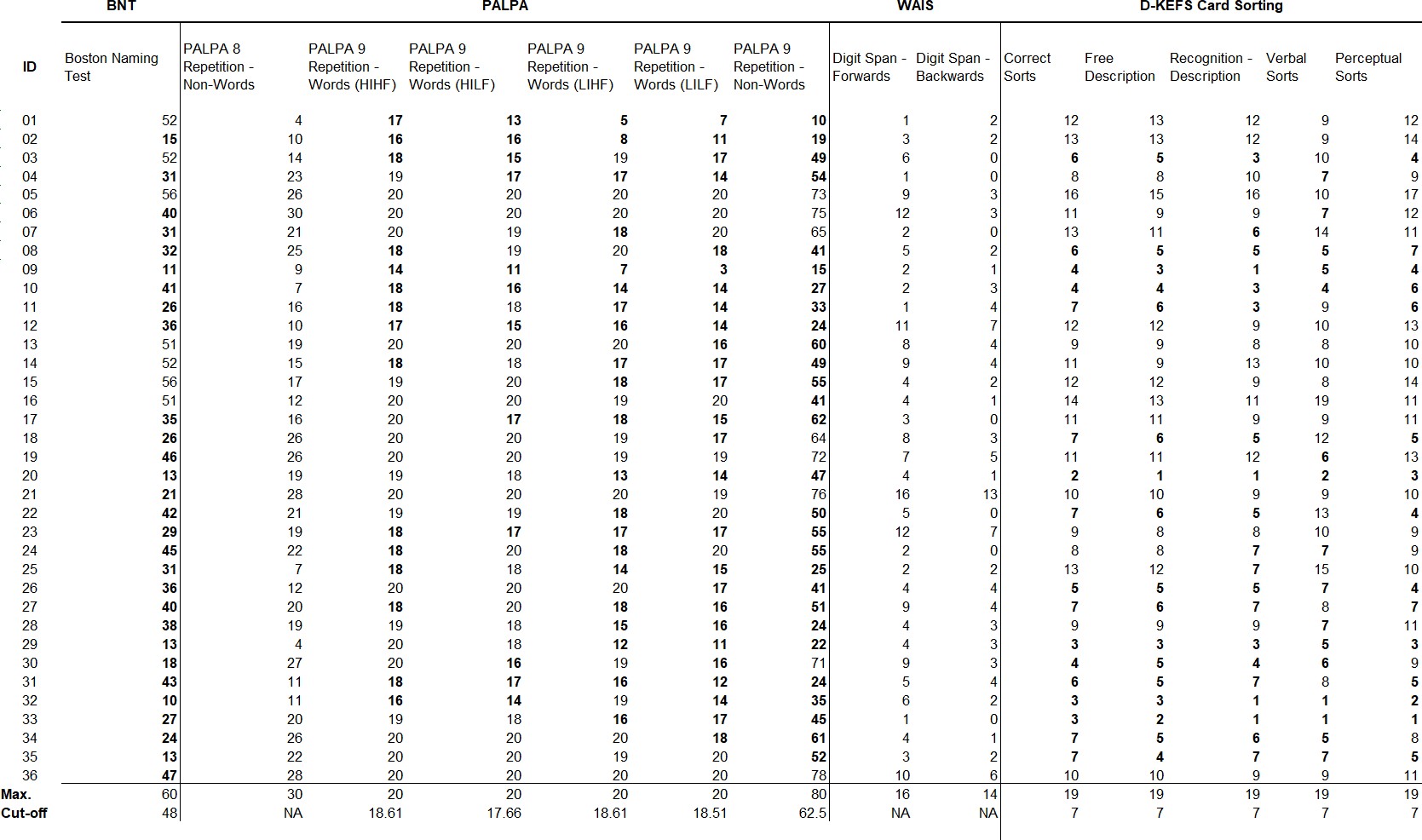


**Supplementary Table 1 (cont.) Participants’ scores on the behavioural assessments**


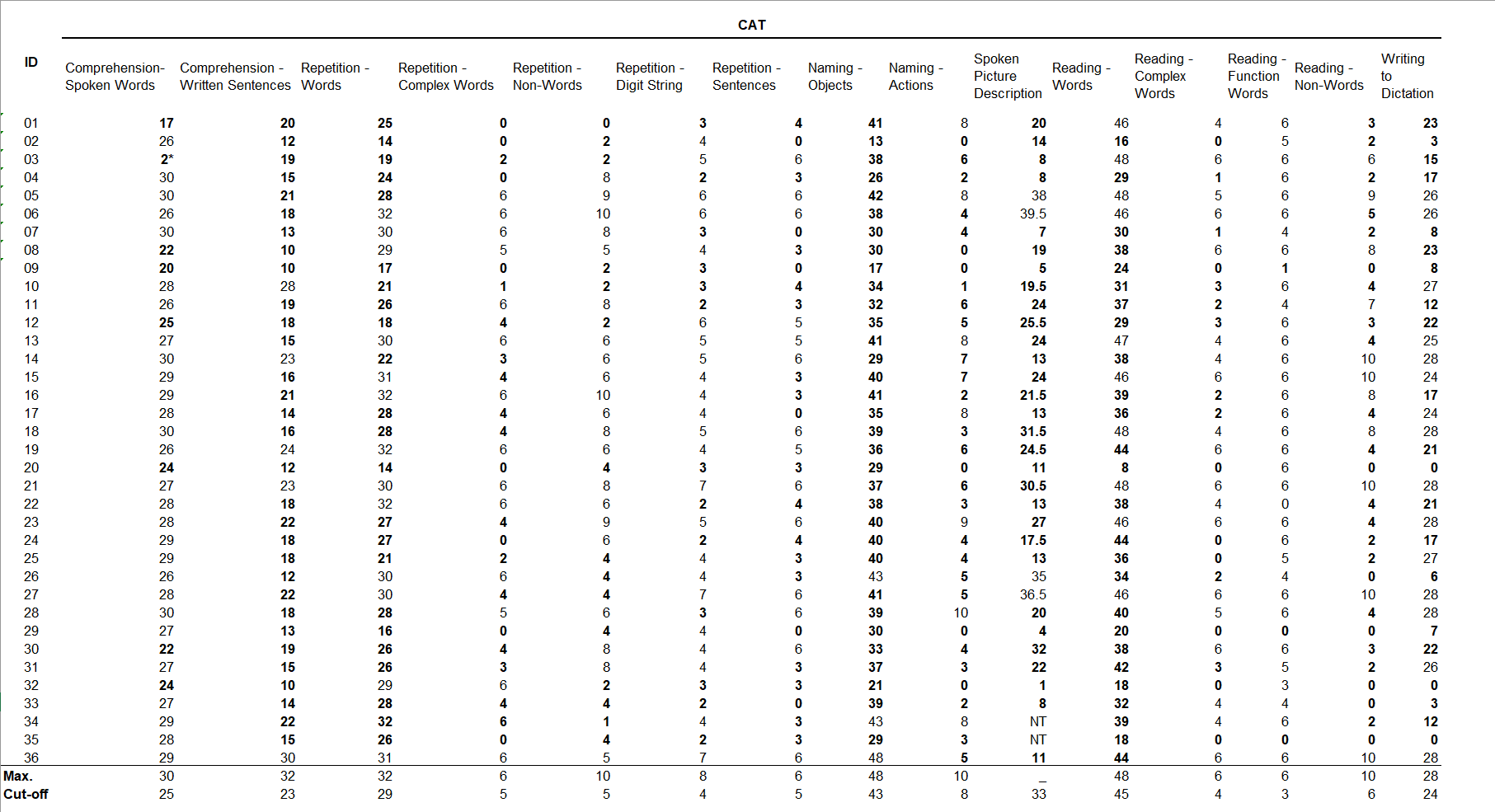


This table displays individual participants’ scores on the behavioural assessments that loaded on factors extracted from the rotated PCA. Scores below cut-off shown in bold. PALPA = Psycholinguistic Assessments of Language Processing in Aphasia; LILF = Low Intelligibility Low Frequency, LIHF = Low Intelligibility High Frequency, HIHF = High Intelligibility High Frequency, HILF = High Intelligibility Low Frequency. CAT = Comprehensive Aphasia Test. DKEFS = Delis-Kaplan Executive Function System. WAIS = Wechsler Adult Intelligence Scale. NA= data not available; NT= not tested. *Participant ID 03 CAT spoken words comprehension subtest terminated after 4 test items – reason not documented – functionally, speech comprehension was reported as good.
